# Screening of disorders associated with osteosarcoma by integrated network analysis

**DOI:** 10.1042/BSR20190235

**Published:** 2019-05-21

**Authors:** Yongfeng Dou, Kai Zhu, Zhaozhong Sun, Xiaopeng Geng, Qingmin Fang

**Affiliations:** Department of Orthopaedic, Binzhou Medical University Hospital, Binzhou 256603, Shandong Province, China

**Keywords:** Association phenotype analysis, dysfunction module, disordered molecules, gene expression, osteosarcoma typing

## Abstract

Osteosarcoma is a common malignant bone tumor in children and adolescents under the age of 20. However, research on the pathogenesis and treatment of osteosarcoma is still insufficient. In the present study, based on gene-phenotype correlation network, an analysis was performed to screen disorders related to osteosarcoma. First, we analyzed the differential expression of osteosarcoma in two groups according to different types of osteosarcoma and screened the differentially expressed genes (DEGs) related to osteosarcoma. Further, these DEG coexpression modules were obtained. Finally, we identified a series of regulatory factors, such as endogenous genes, transcription factors (TFs), and ncRNAs, which have potential regulatory effects on osteosarcoma, based on the prediction analysis of related network of gene phenotypes. A total of 3767 DEGs of osteosarcoma were identified and clustered them into 20 osteosarcoma-related dysfunction modules. And there were 38 endogenous genes (including ARF1, HSP90AB1, and TUBA1B), 53 TFs (including E2F1, NFKB1, and EGR1), and 858 ncRNAs (including MALAT1, miR-590-3p, and TUG1) were considered as key regulators of osteosarcoma through a series of function enrichment analysis and network analysis. Based on the results of the present study, we can show a new way for biologists and pharmacists to reveal the potential molecular mechanism of osteosarcoma typing, and provide valuable reference for different follow-up treatment options.

## Introduction

Osteosarcoma is a common primary malignant bone tumor. It is also the most common primary bone tumor in children and adolescents under 20 years old. It is also the third most common cancer in childhood and adolescence [1–3]. As a malignant tumor, osteosarcoma mainly affects long bones, but may also involve other bones in the body. It has a bimodal distribution and will reach its peak in the second decade of life and adulthood [4]. On the one hand, due to the lack of lymphatic system in the skeleton, metastatic spread in bone and sarcoma is completely hematogenous. The most common sites are lung and bone [5]. On the other hand, osteosarcoma is considered to be a highly vascularized bone tumor, which mainly enters the lung through early metastatic diffusion of intratumoral blood vessels [6]. Osteosarcoma belongs to primary bone tumors. Its recurrence and metastasis potential may be due to cell subsets with stem cell-like characteristics, which maintain the regeneration ability of the whole tumor. Osteosarcoma is associated with the most common genetic abnormalities in human cancers [7]. Contrary to other malignant tumors, many genetic and environmental factors can lead to the development of osteosarcoma. Therefore, osteosarcoma is defined phenotypically rather than molecularly [8,9]. Osteosarcoma is a highly hereditary and unstable tumor with a high incidence. Because of local recurrence, metastasis, and chemotherapy resistance, it has a poor prognosis [10–12]. Despite significant improvements in treatment strategies in recent years, for most patients with metastatic or recurrent osteosarcoma, treatment outcomes are still poor [13]. Studies have shown that osteosarcoma is an invasive cancer in the skeletal system, in which altered oncogenes and tumor suppressor genes can participate in tumor cell migration, angiogenesis, cell apoptosis, and proliferation [14].

Although osteosarcoma is a rare malignant tumor, it is listed as one of the main causes of cancer-related death in the pediatric age group [15]. Therefore, in recent years, many biologists and pharmacists have carried out a series of in-depth studies on the effective treatment of osteosarcoma. The data show that the optimal treatment for osteosarcoma patients is neoadjuvant chemotherapy followed by complete surgical resection and adjuvant chemotherapy [2]. The treatment of osteosarcoma now includes systemic chemotherapy and local control surgery, multidrug chemotherapy, and active surgical techniques, which to some extent improves the survival rate of patients [16,17]. Hornicek et al. have shown that EZH2 is essential for the growth and metastasis of osteosarcoma, and epigenetic therapy targetting EZH2 through specific inhibitors pharmacology can constitute a new method for the treatment of osteosarcoma [12]. It is reported that miRNA-27a can promote the proliferation, migration, and invasion of human osteosarcoma cells. Therefore, miRNA-27a can be used as a non-invasive diagnostic and prognostic biomarker for osteosarcoma patients [18]. In addition, the down-regulation of miRNA-152 can also be considered as a predictor of diagnosis and prognosis in osteosarcoma patients [19]. As a tumor suppressor gene, inactivation of NPRL2 contributes to the development of tumors. The expression of NPRL2 is negatively correlated with the survival rate of osteosarcoma patients, and it has important value as a prognostic factor of osteosarcoma [20]. At present, the best treatment of osteosarcoma includes multidrug chemotherapy and active surgical removal of all the affected parts of the disease. This conclusion has also been confirmed by numerous experimental results [21].

Here, based on the gene-phenotype correlation network, we propose a comprehensive analysis to screen the disorders associated with osteosarcoma typing. We have predicted potential disorder molecules associated with disease, which not only provides a new insight into the typing and treatment of osteosarcoma but also provides rich resources and guidance for biologists to further design experiments

## Materials and methods

### Differential expression analysis

We collected the expression microarray dataset of osteosarcoma (GSE94805) from NCBI Gene Expression Omnibus database [22]. Then, differentially expressed genes (DEGs) of normal versus stationary osteosarcoma and normal versus senile osteosarcoma were identified with R language limma package [23] (*P*<0.05).

### Coexpression analysis

First, DEGs shared by the two DEG sets were screened and then coexpression network analysis was performed on these genes expression profiles with weighted gene coexpression network analysis [24] to explore coexpression modules in osteosarcoma. The weighted value of correlation coefficient is used to calculate the correlation coefficient between any two genes (Pearson coefficient) by taking the N power of the correlation coefficient. The connection between genes in the network obeys scale-free networks, which makes the algorithm more biologically meaningful. Finally, a hierarchical clustering tree is constructed by the correlation coefficient between genes. Different branches of the clustering tree represent different gene modules, and different colors represent different modules.

### Gene function annotation

Exploring the role of gene is often an effective means to study the molecular mechanism of disease, and the function and pathway module genes involving in are helpful for characterizing the effect of gene module on the occurrence and development of disease. Therefore, we used R language clusterProfiler package [25] to carry out GO function (*P* value cutoff = 0.01; qvalue cutoff = 0.01) and KEGG pathway (*P* value cutoff = 0.05; qvalue cutoff = 0.2) enrichment analysis, respectively.

### Prediction of transcription factors and ncRNAs regulating modules

First, all human transcription factor (TF)-target data and human ncRNA-protein data (score > 0.5) were downloaded from TRRUST V2 database [26] and RAID 2.0 database [27], respectively. Furthermore, pivot analysis based on these interaction data was performed to predict the regulatory relationships between TFs, ncRNAs and modules. Pivot analysis refers to searching regulators with at least two interactors in module, and the number of interactors was verified to be significant using the hypergeometric test (*P* value < 0.01). Such regulators were thought be key regulators significantly regulating modules.

### Patient and blood samples

All blood samples were confirmed by experienced pathologists and informed consents were obtained from all patients. Human tissue samples were collected according to the International Ethical Guidelines for Biomedical Research involving human and subjects. This research was approved by the Orthopaedic Department of Binzhou Medical University Hospital and carried out in line with the regulations of the Binzhou Medical University Hospital.

### Verification of key genes by qPCR

Specifically, total RNA in the blood was extracted and transcribed into cDNA with a reverse transcription kit and qPCR reaction was conducted with the SYBR qPCR Detection Kit. The qPCR program began the initial 3-min denaturation step at 95°C to stimulate the hot-start iTaqTM DNA polymerase, followed by 45 cycles of denaturation at 95°C for 10 s, and annealing and extension at 60°C for 45 s. The internal reference genes were β-actin and U6.

## Result

### DEG in osteosarcoma

To screen out potential dysregulated molecules that are closely related to the occurrence and development of osteosarcoma, we identified DEGs of normal versus stationary osteosarcoma (5669 DEGs) and normal versus senile osteosarcoma (8346 DEGs) based on the expression microarray dataset of osteosarcoma. Finally, a total of 3767 DEGs shared by the two DEG sets were obtained for further analysis (Supplementary Table S1).

### Coexpression modules of DEGs in osteosarcoma

**Figure 1:**
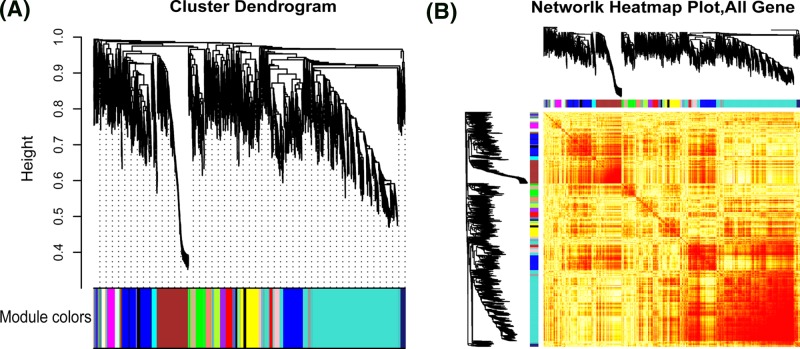
Clustering module of coexpression relations for osteosarcoma-related genes (**A**) According to the coexpression relationship of DEGs, 20 modules are clustered, one color represents one module. (**B**) Thermogram of modular gene expression in samples. Osteosarcoma-related genes are expressed in groups intuitively in disease samples.

**Figure 2:**
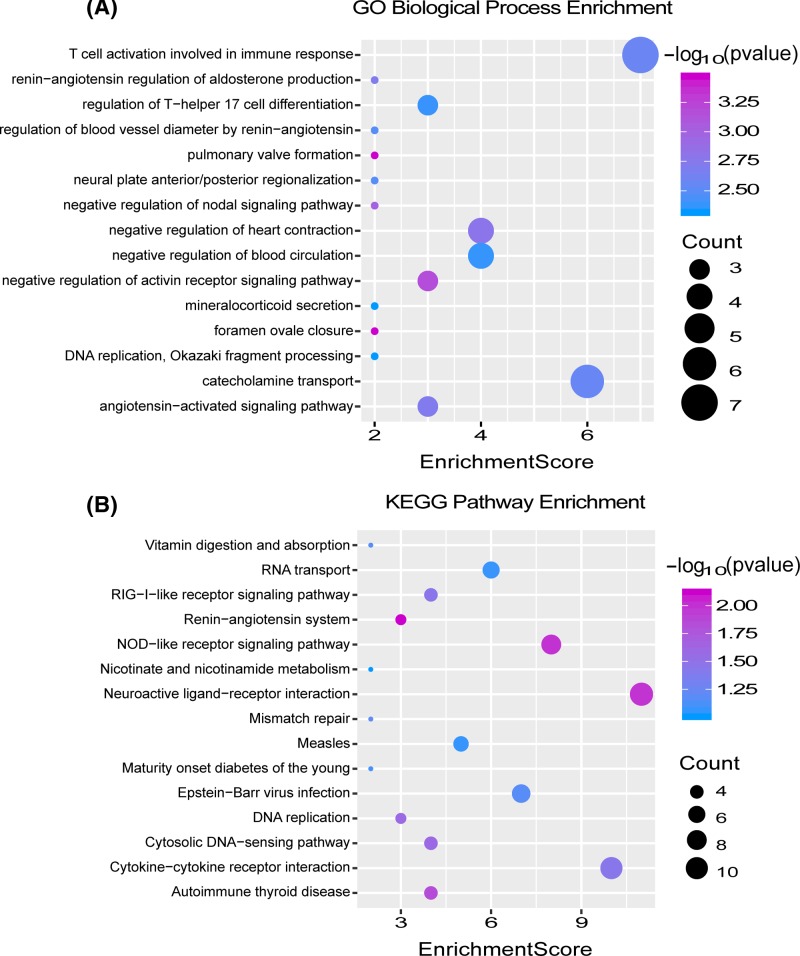
Modular genes are involved in biological functions and signaling pathways (excerpts) (**A**) GO functional enrichment analysis involving module genes. The darker the color, the stronger the significance of enrichment. The larger the circle, the larger the proportion of module genes in GO functional entry genes. (**B**) Enrichment analysis of KEGG pathway involving modular genes. The darker the color, the stronger the significance of enrichment. The larger the circle, the larger the proportion of module genes to KEGG pathway entry genes.

To further investigate the role of DEGs in osteosarcoma, we first performed coexpression analysis based on the expression of 3767 DEGs. A total of 20 coexpression modules were excavated as dysfunction modules of osteosarcoma, involving 3757 DEGs (Figure 1). Genes can influence the occurrence and development of diseases through their own molecular functions (MF) and involved biological processes (BPs). Module as a set of genes will play more significant role in the pathogenesis of disease. Further, the GO function and KEGG pathway enrichment were performed on all module genes. The analysis identified 7433 MF entries, 5274 cell component entries, 4267 BP entries, and 2477 KEGG pathway entries (Figure 2, Supplementary Table S2). In addition, we analyzed network connectivity based on dysfunction module and identified 38 key endogenous genes, including ARF1, HSP90AB1, and TUBA1B.

### Key TFs and ncRNAs significantly regulating modules

**Figure 3:**
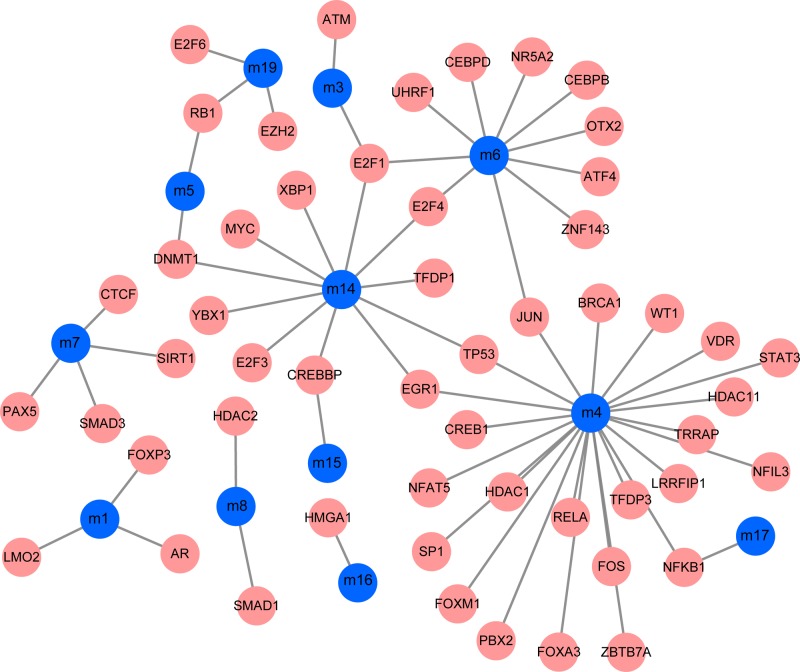
The ncRNA regulatory network of osteosarcoma is represented by red squares and purple squares.

Transcriptional regulation of genes is key regulation in the occurrence and development of diseases, and TFs are an important factor in this process. In the present study, we performed pivot analysis to predict key TFs significantly regulating modules based on TF-gene relationships. The results identified 53 TFs and 63 TF-module interaction pairs (Figure 3, Supplementary Table S3). Statistical analysis showed that E2F1 had significant regulatory relationships with the three dysfunction modules, both NFKB1 and EGR1 regulate the two dysfunction modules.

**Figure 4:**
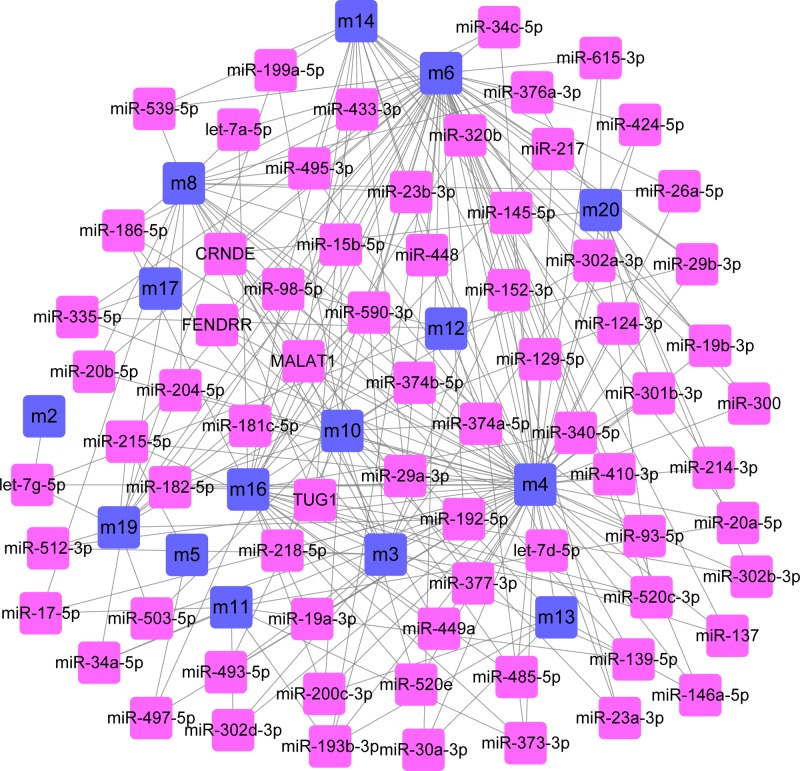
The regulatory network of osteosarcoma TFs to dysfunction modules is shown Blue circle represents the module and pink circle represents the corresponding TFs.

**Figure 5:**
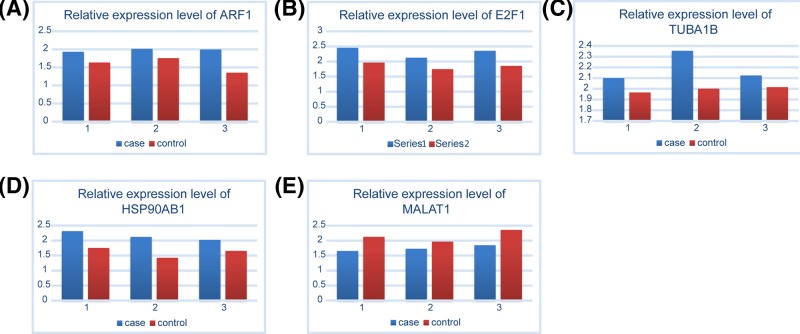
The relative expression level of ARF1, HSP90AB1, TUBA1B, E2F1, and MALAT1

Furthermore, ncRNAs are important regulators of post-transcriptional regulation. Similarly, pivot analysis was performed for key ncRNAs based on relationships between ncRNAs and genes. We have obtained 858 ncRNAs that have significant regulatory effects on modules, involving 1160 ncRNA-module interaction pairs (Figure 4, Supplementary Table S4). Statistical analysis of the results showed that MALAT1 had a regulatory effect on the seven dysfunction modules, miR-590-3p and TUG1 interacted with six dysfunctional modules. These key TFs/ncRNAs may participate in the process of osteosarcoma through regulating disease-related dysfunction modules. Furthermore, the expression level of key genes was verified by qPCR (Figure 5). We found that the expression trend of key genes was consistent with the previous results.

## Discussion

Osteosarcoma is the most common primary bone tumor in children and adolescents, and the prognosis of advanced osteosarcoma patients with metastatic evidence is poor [28]. Pathological interpretation of malignant bone tumors is one of the most challenging areas in surgical pathology, so even if primary osteosarcoma is uncommon, it also shows significant morphological heterogeneity and has a wide range of biological characteristics [29]. Although in recent years biomedical scientists have conducted in-depth studies on the pathogenesis and treatment mechanism of osteosarcoma, there is still a lack of systematic and in-depth exploration of key disorders in the classification of osteosarcoma. In order to further understand the pathogenesis of osteosarcoma, we have integrated abundant resources to conduct a variety of analysis of osteosarcoma, such as gene differential analysis, gene coexpression analysis, transcriptional, and post-transcriptional regulation analysis.

In order to explore the mechanism of osteosarcoma potential pathogenic genes, we first analyzed the genes expression profiles of osteosarcoma, obtained 3767 DEGs, and clustered them into 20 coexpression modules. Subsequently, in view of the results of enrichment analysis, we found that genes in the 20 modules obtained from osteosarcoma mainly participate in nuclear chromosome segregation, microtubule organizing center part and G2/M transition of mitotic cell cycle, and other functional pathways. In addition, we identified 53 TFs that significantly regulating these 20 modules, involving 63 TF-module interaction pairs. It was found that E2F1 had a significant regulatory relationship with the three dysfunction modules and was considered as a key regulation molecule in the process of osteosarcoma. This conclusion has also been confirmed in Zhang and Ding’s research. E2F1 is identified as a new regulator of osteogenic differentiation of osteosarcoma cells induced by ATRA. Meanwhile, E2F1 level has also been found to be associated with poor prognosis of osteosarcoma patients [30,31]. On the other hand, both NFKB1 and EGR1 have been found to regulate the two dysfunction modules, which may also be key regulators involved in the pathogenesis of osteosarcoma. The NF-κB TF, which activates the transcription of genes that regulate a variety of fundamental BPs, including immune response, cell survival, and development [32,33]. NF-κB is significantly involved in the development of breast cancer and other cancers [34]. Nuclear factor-κB1 (NFκB1) gene plays an important role in the pathogenesis of osteosarcoma, which indicates that osteosarcoma is closely related to the gene polymorphism of NFKB1 [35]. According to Matsunoshita et al., EGR1 can be used as a tumor promoter or inhibitor. Experiments show that the strong expression of EGR1 can prevent osteosarcoma cells from migrating into blood vessels, thus inhibiting osteosarcoma to a certain extent [36].

In addition, ncRNA has been considered to be an important regulator of disease occurrence and development, especially inflammatory-related diseases [37]. We conduct pivot analysis based on the targetting relationship between ncRNA and genes. The predicted results show that 858 ncRNAs have significant regulatory effects on modules, including 1160 ncRNA-module interaction pairs. On the one hand, MALAT1 plays an important role in regulating seven dysfunction modules. A number of studies have shown that MALAT1 has carcinogenic effect in osteosarcoma tumorigenesis and metastasis, so it can be considered that MALAT1 may be a promising therapeutic target for osteosarcoma patients [38,39]. On the other hand, miRNAs-590-3p and TUG1 also play an important role in the regulation of six dysfunctional modules, and also play an important role in module dysfunction. MiR-590-3p is an anticancer miRNA, which can inhibit the proliferation and metastasis of osteosarcoma cells [40]. TUG1 knockdown can also inhibit the proliferation, migration, and invasion of osteosarcoma cells and promote cell apoptosis. It can be found that TUG1 knockdown plays an important role in the development of osteosarcoma [41]. At the same time, we also screened a series of genes with the greatest connectivity as the internal gene of the module. A total of 38 endogenous genes were obtained, which may represent potential key regulators of osteosarcoma typing. Statistical analysis of these endogenous genes showed that some of them could be identified as having a regulatory role in the pathogenesis of osteosarcoma. Amongst them, the discovery of ARF1 is of great significance for improving the diagnosis of osteosarcoma [42].

A series of regulatory factors predicted in the present study have certain regulatory effects on the potential pathogenesis of osteosarcoma in varying degrees. However, in addition to the above key factors, other unmentioned ncRNA and transcription factors may play a role in the pathogenesis of osteosarcoma, which need further exploration. Overall, our study is based on the results of phenotypic correlation network analysis, and screening guides the identification of key disorders in osteosarcoma typing. This will not only provide a new way for biologists and pharmacists to study the pathogenesis of osteosarcoma but also provide valuable reference for their follow-up treatment.

## Supporting information

**Supplemental Table S1:** 

**Supplemental Table S2:** 

**Supplemental Table S3:** 

**Supplemental Table S4:** 

## Abbreviations

ATRA: all-trans retinoic acid
DEG: differentially expressed gene
GO: GO Ontology
KEGG: Kyoto Encyclopedia of Genes and Genomes
MF: molecular function
ncRNA: non-coding RNA
qPCR: quantitative real-time polymerase chain reaction
TF: transcription factor

## References

[1] Chen R., Huang L.H., Gao Y.Y., Yang J.Z. and Wang Y. (2018) Identification of differentially expressed genes in MG63 osteosarcoma cells with drugresistance by microarray analysis. Mol. Med. Rep. 10.3892/mmr.2018.9774 PMC6390052 30569145

[2] Karadurmus N., Sahin U., Bahadir Basgoz B. and Demirer T. (2018) Is there a role of high dose chemotherapy and autologous stem cell transplantation in the treatment of Ewing’s sarcoma and osteosarcomas? J. BUON 23, 1235–1241 30570842

[3] Bernardini G., Laschi M., Geminiani M. and Santucci A. (2014) Proteomics of osteosarcoma. Exp. Rev. Proteomics 11, 331–343 10.1586/14789450.2014.900445 24654989

[4] Moore D.D. and Luu H.H. (2014) Osteosarcoma. Cancer Treat. Res. 162, 65–92 10.1007/978-3-319-07323-1_4 25070231

[5] Goyal S. and Julka P.K. (2017) Recurrent osteosarcoma with calcified liver metastases: uncommon development of a common disease. J. Cancer Res. Ther. 13, 139–141 10.4103/0973-1482.148672 28508848

[6] Kunz P., Fellenberg J., Moskovszky L., Sapi Z., Krenacs T., Machado I. (2015) Improved survival in osteosarcoma patients with atypical low vascularization. Ann. Surg. Oncol. 22, 489–496 10.1245/s10434-014-4001-2 25155396

[7] Tamari K., Hayashi K., Ishii H., Kano Y., Konno M., Kawamoto K. (2014) Identification of chemoradiation-resistant osteosarcoma stem cells using an imaging system for proteasome activity. Int. J. Oncol. 45, 2349–2354 10.3892/ijo.2014.2671 25269626

[8] Gorlick R. and Khanna C. (2010) Osteosarcoma. J. Bone Miner. Res. 25, 683–691 10.1002/jbmr.77 20205169

[9] Gorlick R. (2009) Current concepts on the molecular biology of osteosarcoma. Cancer Treat. Res. 152, 467–478 10.1007/978-1-4419-0284-9_27 20213409

[10] Zhou G., Shi X., Zhang J., Wu S. and Zhao J. (2013) MicroRNAs in osteosarcoma: from biological players to clinical contributors, a review. J. Int. Med. Res. 41, 1–12 10.1177/0300060513475959 23569124

[11] Du X., Yang J., Yang D., Tian W. and Zhu Z. (2014) The genetic basis for inactivation of Wnt pathway in human osteosarcoma. BMC Cancer 14, 450 10.1186/1471-2407-14-450 24942472 PMC4074405

[12] Sun R., Shen J., Gao Y., Zhou Y., Yu Z., Hornicek F. (2016) Overexpression of EZH2 is associated with the poor prognosis in osteosarcoma and function analysis indicates a therapeutic potential. Oncotarget 7, 38333–38346 27223261 10.18632/oncotarget.9518 PMC5122393

[13] Li Z., Dou P., Liu T. and He S. (2017) Application of long noncoding RNAs in osteosarcoma: biomarkers and therapeutic targets. Cell. Physiol. Biochem. 42, 1407–1419 10.1159/000479205 28715796

[14] Zhou W., Hao M., Du X., Chen K., Wang G. and Yang J. (2014) Advances in targeted therapy for osteosarcoma. Discov. Med. 17, 301–307 24979249

[15] Botter S.M., Neri D. and Fuchs B. (2014) Recent advances in osteosarcoma. Curr. Opin. Pharmacol. 16, 15–23 10.1016/j.coph.2014.02.002 24632219

[16] Anderson M.E. (2016) Update on survival in osteosarcoma. Orthop. Clin. North Am. 47, 283–292 10.1016/j.ocl.2015.08.022 26614941

[17] Fenger J.M., London C.A. and Kisseberth W.C. (2014) Canine osteosarcoma: a naturally occurring disease to inform pediatric oncology. ILAR J. 55, 69–85 10.1093/ilar/ilu009 24936031

[18] Tang J., Zhao H., Cai H. and Wu H. (2015) Diagnostic and prognostic potentials of microRNA-27a in osteosarcoma. Biomed. Pharmacother. 71, 222–226 10.1016/j.biopha.2015.01.025 25960240

[19] Wang N.G., Wang D.C., Tan B.Y., Wang F. and Yuan Z.N. (2015) Down-regulation of microRNA152 is associated with the diagnosis and prognosis of patients with osteosarcoma. Int. J. Clin. Exp. Pathol. 8, 9314–9319 26464682 PMC4583914

[20] Gao Y., Wang J. and Fan G. (2012) NPRL2 is an independent prognostic factor of osteosarcoma. Cancer Biomark. 12, 31–36 10.3233/CBM-120290 23321467 PMC13016367

[21] Chou A.J., Geller D.S. and Gorlick R. (2008) Therapy for osteosarcoma: where do we go from here? Paediatr. Drugs 10, 315–327 10.2165/00148581-200810050-00005 18754698

[22] Barrett T., Wilhite S.E., Ledoux P., Evangelista C., Kim I.F., Tomashevsky M. (2013) NCBI GEO: archive for functional genomics data sets–update. Nucleic. Acids Res. 41, D991–D995 10.1093/nar/gks1193 23193258 PMC3531084

[23] Ritchie M.E., Phipson B., Wu D., Hu Y., Law C.W., Shi W. (2015) limma powers differential expression analyses for RNA-sequencing and microarray studies. Nucleic. Acids Res. 43, e47 10.1093/nar/gkv007 25605792 PMC4402510

[24] Langfelder P. and Horvath S. (2008) WGCNA: an R package for weighted correlation network analysis. BMC Bioinformatics 9, 559 10.1186/1471-2105-9-559 19114008 PMC2631488

[25] Yu G., Wang L.G., Han Y. and He Q.Y. (2012) clusterProfiler: an R package for comparing biological themes among gene clusters. OMICS 16, 284–287 10.1089/omi.2011.0118 22455463 PMC3339379

[26] Han H., Cho J.W., Lee S., Yun A., Kim H., Bae D. (2018) TRRUST v2: an expanded reference database of human and mouse transcriptional regulatory interactions. Nucleic. Acids Res. 46, D380–D386 10.1093/nar/gkx1013 29087512 PMC5753191

[27] Yi Y., Zhao Y., Li C., Zhang L., Huang H., Li Y. (2017) RAID v2.0: an updated resource of RNA-associated interactions across organisms. Nucleic. Acids Res. 45, D115–D118 10.1093/nar/gkw1052 27899615 PMC5210540

[28] Brown H.K., Tellez-Gabriel M. and Heymann D. (2017) Cancer stem cells in osteosarcoma. Cancer Lett. 386, 189–195 10.1016/j.canlet.2016.11.019 27894960

[29] Rosenberg A.E. (2017) Bone sarcoma pathology: diagnostic approach for optimal therapy. Am. Soc. Clin. Oncol. Educ. Book 37, 794–798 10.14694/EDBK_174697 28561653

[30] Zhang L., Zhou Q., Zhang N., Li W., Ying M., Ding W. (2014) E2F1 impairs all-trans retinoic acid-induced osteogenic differentiation of osteosarcoma via promoting ubiquitination-mediated degradation of RARalpha. Cell Cycle 13, 1277–1287 10.4161/cc.28190 24608861 PMC4049964

[31] Wang Z., Sun X., Bao Y., Mo J., Du H., Hu J. (2017) E2F1 silencing inhibits migration and invasion of osteosarcoma cells via regulating DDR1 expression. Int. J. Oncol. 51, 1639–1650 10.3892/ijo.2017.4165 29039472 PMC5673022

[32] Irelan J.T., Murphy T.J., DeJesus P.D., Teo H., Xu D., Gomez-Ferreria M.A. (2007) A role for IkappaB kinase 2 in bipolar spindle assembly. Proc. Natl Acad. Sci. U.S.A. 104, 16940–16945 10.1073/pnas.0706493104 17939994 PMC2040438

[33] Correa R.G., Matsui T., Tergaonkar V., Rodriguez-Esteban C., Izpisua-Belmonte J.C. and Verma I.M. (2005) Zebrafish IkappaB kinase 1 negatively regulates NF-kappaB activity. Curr. Biol. 15, 1291–1295 10.1016/j.cub.2005.06.023 16051172

[34] Shin E.M., Hay H.S., Lee M.H., Goh J.N., Tan T.Z., Sen Y.P. (2014) DEAD-box helicase DP103 defines metastatic potential of human breast cancers. J. Clin. Invest. 124, 3807–3824 10.1172/JCI73451 25083991 PMC4151228

[35] Li X., Zhang C., Qiao W., Zhou X. and Sun M. (2015) NFKB1 -94ins/del ATTG polymorphism increases osteosarcoma risk in a Chinese Han population. Int. J. Clin. Exp. Med. 8, 1420–1423 25785149 PMC4358604

[36] Matsunoshita Y., Ijiri K., Ishidou Y., Nagano S., Yamamoto T., Nagao H. (2011) Suppression of osteosarcoma cell invasion by chemotherapy is mediated by urokinase plasminogen activator activity via up-regulation of EGR1. PLoS ONE 6, e16234 10.1371/journal.pone.0016234 21283769 PMC3024416

[37] Chew C.L., Conos S.A., Unal B. and Tergaonkar V. (2018) Noncoding RNAs: master regulators of inflammatory signaling. Trends Mol. Med. 24, 66–84 10.1016/j.molmed.2017.11.003 29246760

[38] Liu K., Huang J., Ni J., Song D., Ding M., Wang J. (2017) MALAT1 promotes osteosarcoma development by regulation of HMGB1 via miR-142-3p and miR-129-5p. Cell Cycle 16, 578–587 10.1080/15384101.2017.1288324 28346809 PMC5384591

[39] Cai X., Liu Y., Yang W., Xia Y., Yang C., Yang S. (2016) Long noncoding RNA MALAT1 as a potential therapeutic target in osteosarcoma. J. Orthop. Res. 34, 932–941 10.1002/jor.23105 26575981

[40] Wang W.T., Qi Q., Zhao P., Li C.Y., Yin X.Y. and Yan R.B. (2018) miR-590-3p is a novel microRNA which suppresses osteosarcoma progression by targeting SOX9. Biomed. Pharmacother. 107, 1763–1769 10.1016/j.biopha.2018.06.124 30257395

[41] Cao J., Han X., Qi X., Jin X. and Li X. (2017) TUG1 promotes osteosarcoma tumorigenesis by upregulating EZH2 expression via miR-144-3p. Int. J. Oncol. 51, 1115–1123 10.3892/ijo.2017.4110 28902349 PMC5592872

[42] Olstad O.K., Gautvik V.T., Reppe S., Rian E., Jemtland R., Ohlsson C. (2003) Molecular heterogeneity in human osteosarcoma demonstrated by enriched mRNAs isolated by directional tag PCR subtraction cloning. Anticancer Res. 23, 2201–2216 12894494

